# Artificial Neuron Based on the Bloch-Point Domain Wall in Ferromagnetic Nanowires

**DOI:** 10.3390/ma17102425

**Published:** 2024-05-17

**Authors:** Carlos Sánchez, Diego Caso, Farkhad G. Aliev

**Affiliations:** 1 Departamento de Física de la Materia Condensada C03, Universidad Autónoma de Madrid, 28049 Madrid, Spain; carlos.sanchezcruz@estudiante.uam.es (C.S.); diego.caso@uam.es (D.C.); 2 Instituto Nicolás Cabrera (INC), Universidad Autónoma de Madrid, 28049 Madrid, Spain; 3 Condensed Matter Physics Institute (IFIMAC), Universidad Autónoma de Madrid, 28049 Madrid, Spain

**Keywords:** nanowire, bloch points, artificial neuron

## Abstract

Nanomagnetism and spintronics are currently active areas of research, with one of the main goals being the creation of low-energy-consuming magnetic memories based on nanomagnet switching. These types of devices could also be implemented in neuromorphic computing by crafting artificial neurons (ANs) that emulate the characteristics of biological neurons through the implementation of neuron models such as the widely used leaky integrate-and-fire (LIF) with a refractory period. In this study, we have carried out numerical simulations of a 120 nm diameter, 250 nm length ferromagnetic nanowire (NW) with the aim of exploring the design of an artificial neuron based on the creation and destruction of a Bloch-point domain wall. To replicate signal integration, we applied pulsed trains of spin currents to the opposite faces of the ferromagnetic NW. These pulsed currents (previously studied only in the continuous form) are responsible for inducing transitions between the stable single vortex (SV) state and the metastable Bloch point domain wall (BP-DW) state. To ensure the system exhibits leak and refractory properties, the NW was placed in a homogeneous magnetic field of the order of mT in the axial direction. The suggested configuration fulfills the requirements and characteristics of a biological neuron, potentially leading to the future creation of artificial neural networks (ANNs) based on reversible changes in the topology of magnetic NWs.

## 1. Introduction

The remarkable energy efficiency of the brain in tackling logical and numerical challenges inspired researchers to explore the feasibility of developing novel hardware devices capable of emulating neurons to solve complex practical problems. However, current artificial neurons (ANs) predominantly rely on conventional MOSFET electronics with von Neumann architecture, which imposes limitations due to their high energy consumption and speed constraints. A paradigm shift in hardware design is imperative to enhance energy efficiency and downsize device dimensions.

In recent years, a number of nanoscale devices and materials have been proposed for employment in neuromorphic data processing [1,2], including materials featuring resistive switches, spintronics, and photonic structures (see [3] for a recent review). While joule heating, speed, and scalability are among the main challenges for neuromorphic elements based on resistive switching [4], spin-based data processing could offer scalability, sub-nanosecond speeds, and low dissipation if the operation is mediated by spin waves. Systems utilizing spintronics nanostructures for non-conventional computing currently include magnetic tunnel junctions for neuromorphic [5] or stochastic [6] computing (see [7] for a recent review), spin wave logics [8,9] and lithographically patterned nanowires [10]. In magnetic nanostructures, information can be encoded and processed by using magnetic signals detected through electromagnetic induction or tunneling magnetoresistance and manipulated using spin torque [10].

A great advantage of using ferromagnetic nanowires is that they can be integrated with other nanoscale components and materials to produce complex neuromorphic systems. For example, they could be combined with memristors (particularly those based on magnetic tunnel junctions [11]) to create hybrid neuromorphic architectures leading to novel computing paradigms. Their implementation in nanowire networks (NWNs) is also emerging as a novel class of neuromorphic systems that exploit their distinctive physical properties [12,13]. The manipulation of magnetic textures in nanoscale ferromagnetic devices is one of the most promising routes being currently explored for neuromorphic computing. Some of the ways proposed during the last years to implement neuromorphic hardware by using magnetic nanostructures include magnetic skyrmions [14], magnetic domain walls [15,16], or chiral magnets hosting skyrmions [17].

Here, we introduce an artificial neuron concept centered around the reversible control of a Bloch-point domain wall (BP-DW) in ferromagnetic nanowires (see Refs. [18,19,20,21,22]). We elucidate the methodologies and present experimental trials, demonstrating that the proposed device meets the requisites and characteristics akin to those of biological neurons. We envision that employing short ferromagnetic nanowires as the foundation for hardware could pave the way for the development of artificial neural networks (ANNs) based on reversible topology control. This innovation has the potential to realize low-power ferromagnetic nanowire array chips capable of learning and resolving real-time problems.

## 2. Methods

**Figure 1:**
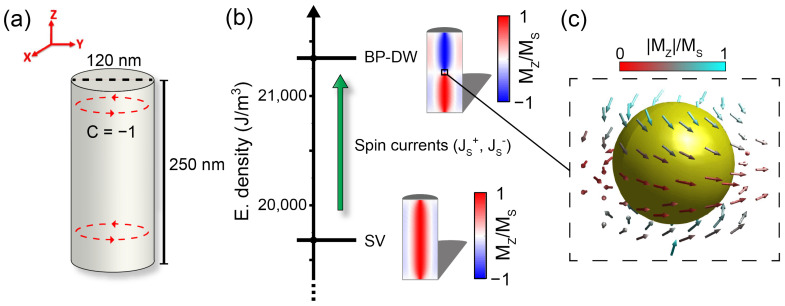
(**a**) Sketch of the cylindrical NW and its orientation with respect to the cartesian coordinate axes, showing the circulation direction of the magnetization around the vortex core. (**b**) Energy scheme of the two metastable states with longitudinal cuts (xz-plane) of the NW showing the SV and BP-DW magnetization configurations. (**c**) 3D magnetization surrounds the BP-DW, centered in the middle of the NW. Colors represent the magnitude of the axial-aligned magnetization.

Previously, Caso et al. [18] demonstrated by means of micromagnetic simulations that the energy gap between the two-level system formed by the stable single vortex (SV) and BP-DW metastable states in short ferromagnetic NWs can be overcome by using either spin currents or frequency tuned magnetic pulses. The main goal of the current study is to numerically investigate the control over the transition between SV and the BP-DW states (see Figure 1b) using a train of spin current pulses with the aim of creating an artificial neuron based on short ferromagnetic nanowires.

### Simulation Details

Micromagnetic simulations were conducted using the open-source software MuMax3 (version 3.10) [23]. The simulated systems consist of individual NWs composed of FeCoCu (Fe_28_Co_67_Cu_5_). A discretization cell size of 1.5×1.5×1.25 nm was chosen, significantly smaller than the exchange length of approximately 3 nm for FeCoCu [24,25]. The cell size is chosen based on the typical scale of the magnetic topological textures that can appear in the system. We chose a smaller discretization in z to better resolve the magnetic topologies in this particular direction. We estimate a DW and vortex core thickness of about 10 nm. The NW diameter was set at 120 nm and its length at 250 nm. This particular length was selected based on previous studies [18], where the system showed a quasi-minima in the energy gap between the NW’s stable states ($\Delta E=5.98\times 10^{-18}$ J) and due to overall computational efficiency reasons, since the minima is at L = 400 nm (see Appendix A). Even though the simulations are carried out at 0 K, the gap between the SV and BP-DW states showed to be robust to a possible thermal magnetic switch at room temperature (ΔE/kT∼1450 at T = 300 K) for this length. Previously studied NWs were either much longer (more than 1 µm in length) or were almost disk-shaped (circular dots with a thickness of much less than 100 nm). We used typical magnetic parameters of ferromagnetic FeCoCu NWs in our simulations [26,27]: $M_{s}=2$ T; $\gamma_{e}=1.759\times 10^{11}$ rad/sT; α=0.01; $A_{ex}=25\times 10^{-12}$ J/m, where M_*s*_ is the saturation magnetization of the NW, $\gamma_{e}$ is the gyromagnetic ratio, and A_*ex*_ and α refer to the exchange stiffness constant and the damping of the NW, respectively. α is reduced to 10^−4^ in the dynamic simulations to better resolve the modes of the system. The NWs axis was set to be parallel to the *z* axis in the cartesian coordinate system (see Figure 1a), i.e., the bases of the NW are placed parallel to the xy plane.

Due to their cylindrical geometry, the NWs display a considerable shape anisotropy, which favors the magnetization to be aligned with the NWs axis direction. Its circular shape also invites the NW magnetization to circulate along the axis. For this reason, the SV state is stable in our system and has a slightly lower energy than the other stable state in the NW, the BP-DW state, as seen in Figure 1b, emulating two states of different membrane potential in our neuron. Figure 1c depicts the vicinity of the Bloch point in our system, corresponding to a singularity enclosed in a head-to-head vortex domain wall. No additional magneto-crystalline anisotropies have been introduced in the simulations.

To reveal the primary spin wave (SW) modes (see Appendix B), we examined the averaged in-plane magnetization of the system using a Fourier transform [28] to transition into the frequency domain. The analysis was conducted following the application of a homogeneous magnetic field pulse of the form:

(1) $${\vec{B}}_{\mathrm{pulse}}=B_{\mathrm{pulse}}\sin c\left(\frac{2\pi t}{t_{0}}\right){\hat{u}}_{y};$$

where B_pulse_ is the amplitude of the applied field pulse (2 mT), and $t_{0}$ denotes the full width at half maximum (FWHM) of the pulse (1 × 10^−12^ s). The pulse is modeled using the sinc(x) function, which demonstrated better performance than a Gaussian function. This choice of pulse shape results in narrower and more well-defined peaks in the spin wave excitation spectrum obtained through Fourier transform analysis.

**Figure 2:**
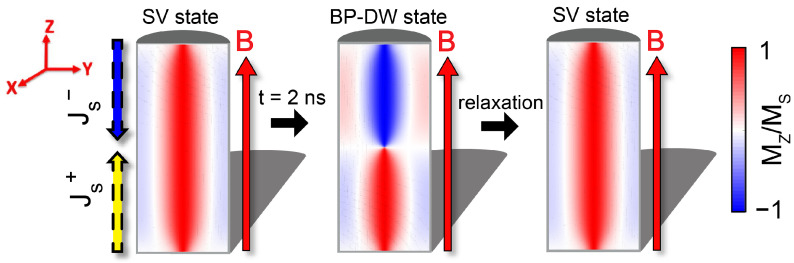
Cross-section of the axial magnetization component M_*z*_/M_*s*_ in the 250 nm long, 120 nm diameter NW. The steps followed in the study are consequently illustrated. First, input pulsed spin currents ($J_{s}^{+}$ and $J_{s}^{-}$) are injected in the SV state on the opposite faces of the NW. After approximately 2 ns, the system enters the BP-DW state and fires. Following this, the pulsed signal is stopped, and the system recovers into its initial SV state with the help of an axial homogeneous magnetic field of B = 1 mT ${\hat{u}}_{z}$.

As previously explored by Caso et al. [18], we examined the transition between the SV and BP-DW states. We claim that this switch fulfills the characteristics of biological neurons under certain field and spin-current conditions. In order to demonstrate that, the initial configuration of the system was set to be an SV state. Afterwards, squared pulsed trains of spin-currents were applied at the opposite faces of the cylinder. These currents are fully polarized (P=−1), and their frequencies are based on those of the SW modes. Furthermore, we have verified the sensitivity of the AN up to a much higher (about 1000 GHz) and much lower (below 1 GHz) frequency in trains of squared pulses. Additionally, a uniform magnetic field in the axial direction ($\vec{B}=1$ mT ${\hat{u}}_{z}$) was introduced to break axial symmetry and facilitate the system’s return to its initial SV configuration after the transition (see Figure 2).

## 3. Proof of Concept of the Artificial Neuron: Results and Discussion

The LIF (Leaky-Integrate-and-Fire) neuron model [29] simulates the behavior of a biological neuron by accumulating incoming synaptic currents (integrate with some leak) until it reaches a threshold, triggering a spike or action potential (fire). Here we investigate the different functionalities of a LIFR neuron in relation to our NW system.

### 3.1. Integrate Functionality

**Figure 3:**
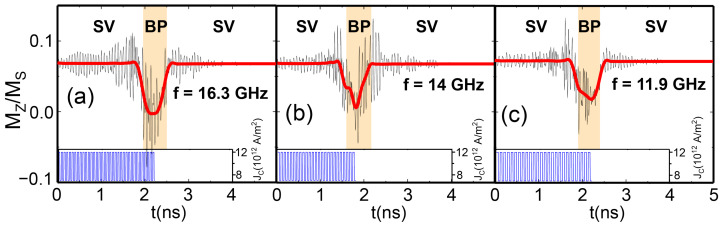
Evolution of the average axial magnetization of the NW for spin current frequencies of (**a**) 16.3 GHz, (**b**) 14 GHz, and (**c**) 11.9 GHz. The insets in both graphs represent the input spin-current pulses by means of the applied current. The smoothed red curves are obtained by means of FFT filtering the high-frequency oscillations.

To be biofidelic, our artificial neuron must exhibit sensitivity to input pulsed signals (integrate functionality). In the context of our NWs, this criterion is unequivocally met when the spin current pulses reach a sufficient amplitude, causing the axial projection of the magnetization in the SV state to oscillate (see Figure 3). The energy barrier between the BP-DW and the SV states is overcome for sufficiently high spin current pulses. Introducing a baseline DC spin current of 7 × $10^{12}$ A/m^2^ along with the AC (i.e., periodic pulse) spin current inputs with an amplitude of 5 × $10^{12}$ A/m^2^, reaching a maximum of 12 × $10^{12}$ A/m^2^, ensures a controlled transition, preventing an erratic behavior in the shift from SV to BP-DW states.

### 3.2. Leak Functionality

**Figure 4:**
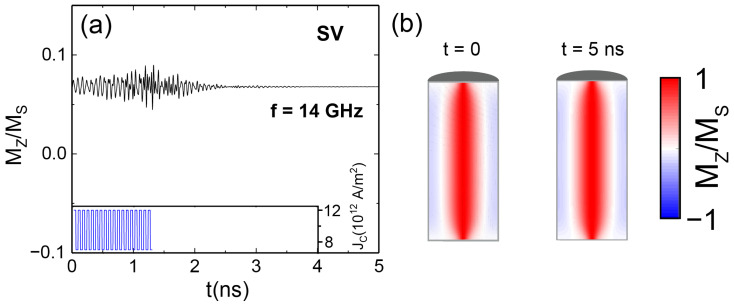
(**a**) Evolution of the average axial magnetization of the NW when applying oscillatory spin currents of frequency 14 GHz and a magnitude of 5 × $10^{12}$ A/m^2^ upon continuous spin currents of magnitude 7 × $10^{12}$ A/m^2^ for 1.3 ns. The inset represents the input spin-current pulses by means of the applied current. (**b**) Cross-sections of the magnetic state configuration in the NW for the initial time and after 5 ns.

The leak functionality in a neuron is fulfilled once there is a passive decay of the membrane potential in the absence of a pulsed input, reflecting the tendency of the membrane in the natural neuron to return to its resting state. Our AN reflects this behavior. Indeed, once the injection of the spin currents is stopped before the fire output, the oscillating AN recovers back into the SV state when sufficient time has passed. Figure 4a shows this behavior, as the input spin current pulses were introduced just in the first 1.3 nanoseconds and then suddenly stopped. We observe the behavior of the axial SV magnetization configuration in the NW for 5 ns, concluding in the disappearance of the small oscillations. Figure 4b displays a comparison of axial magnetization cross-sections of the initial magnetization and the magnetization after the 5 ns when the pulses are applied on the first 1.3 nanoseconds, clearly concluding in the same magnetization state and therefore demonstrating the leak functionality of our AN.

### 3.3. Fire Functionality

Once the accumulation of input pulses is large enough, the NW reaches the BP-DW state, identified with the fire functionality in the artificial neuron, reaching a threshold of axial magnetization in the NW of $M_{z}/M_{s}=0$ (see Figure 3). This activation is a key element for a future neural network based on the proposed NW system, allowing the network to learn and make decisions based on patterns.

**Figure 5:**
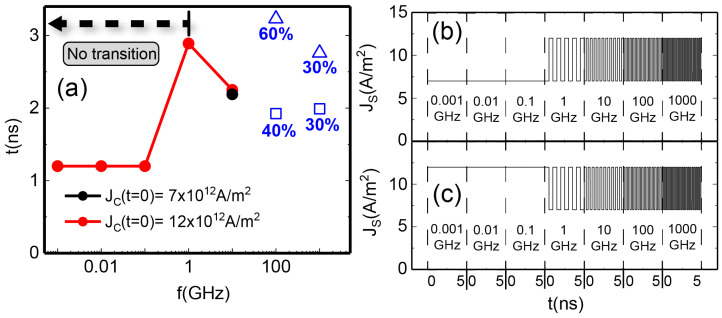
(**a**) Time of transition to the BP-DW state against the frequency of the input spin current pulses in logarithmic scale. In black, the pulses have no component at t=0, and the amplitude of the spin currents starts at 7 × $10^{12}$ A/m^2^. In red, the AC component is initiated at the maximum of 5 × $10^{12}$ A/m^2^, and the spin currents have an initial amplitude of 12 × $10^{12}$ A/m^2^. Blue triangles and squares represent the stochastic nature of the BP stabilization times at high frequencies. (**b**,**c**) depict the profile of the input spin current pulses, starting at t = 0 from 7 × $10^{12}$ A/m^2^ and 12 × $10^{12}$ A/m^2^, respectively, for each of the examined frequencies in panel (**a**). The pulses in (**b**,**c**) extend for 5 ns at each different frequency, surpassing any recorded transition time.

Figure 3 shows the controlled transition into the BP-DW for three different frequencies of the applied spin current pulses. Two of them, 16.3 GHz (Figure 3a) and 11.9 GHz (Figure 3c), correspond to eigenmodes of the system (see Appendix B for the spin wave spectra). On the other hand, 14 GHz (Figure 3b) does not coincide with any eigenmode. In our case, the AN reaches the BP-DW state even if the injected spin current pulses do not precisely align with the eigenfrequency of the system, as shown in Figure 3b for 14 GHz. However, it is mandatory that the frequency of these pulses be within the range of the eigenmode. If the frequency of the pulses is too low or too high, the transition is accomplished only due to the DC spin current baseline. For this type of state transition with only DC spin currents, Caso et al. [18] demonstrated a switching time of ∼4 ns, which is not improved for either very high or very low frequency input pulses (see Figure 5). However, for an input oscillating signal in the GHz range, the switching time is improved to up to 2 ns (see Figure 3), proving the effect of the AC spin currents.

Figure 5 shows the SV to BP-DW transition stimulated by input AC spin current pulses in a broad frequency range. At very low input pulse frequencies (10^−3^ – 10^−1^ GHz), the AC component of the spin currents remains constant for the duration of the simulation until the transition occurs (or if there is no transition, up to at least 5 ns, see Figure 5b,c). In such cases, the switch behaves as if there was only an injection of a DC spin current. Initiating the amplitude of the spin currents at 7 × $10^{12}$ A/m^2^ for t=0, the current density is so low at low frequencies that the system enters into a chaotic behavior before undergoing any transition, as demonstrated in [18]. When the pulses start at the maximum of 5 × $10^{12}$ A/m^2^, the system behaves as if a constant current of 12 × $10^{12}$ A/m^2^ was being applied, transitioning into BP-DW state in 1.2 ns. In Figure 5, it is shown that only in the range of the tenths of GHz a consistent firing time around 2.2 ns is achieved whether the pulses start to behave as sine or as cosine functions. The input AC spin currents are of great importance for the switch since the transition fails to occur with just a baseline of DC spin currents when the baseline is not extremely high and thus very energetically inefficient. It was also checked that a low DC baseline (4 × $10^{12}$ A/m^2^) with an AC input of 5 × $10^{12}$ A/m^2^ did not achieve any transition, demonstrating that both components are helpful in the switch of states.

In Figure 5, we also show the stochastic nature of the system at high input pulse frequencies: for the ranges of 100 GHz and 1000 GHz, the time of transition or even its presence has a strong dependence on the initial conditions of the pulses. To confirm the stochasticity of the system at high frequencies, simulations at these ranges were repeated ten times. In both cases, the amplitude of the spin current pulses at t = 0 was not a factor since the frequency was so high. Remarkably, for 100 GHz, all simulations resulted in the NW entering the BP-DW state, but they were distributed between being stable at 1.9 ns (40% of the time) or 3.2 ns (60% of the time). For 1000 GHz, the transition was stabilized in 60% of cases, 30% in 2 ns, and 30% in 2.8 ns. The remaining 40% of the time, the transition was uncontrolled, and the result was chaotic system behavior. Rather than being considered a drawback, this stochastic nature is also observed in the brain and neurons. Hence, stochasticity becomes a trait that an artificial neuron may possess in order to be implemented in neuromorphic computing [3] and could be leveraged particularly in this high-frequency ranges. However, for tenths of GHz (aligning with the frequency range of the spin wave modes; see Appendix B), the transition becomes more efficient and controlled. This frequency range showcases not only deterministic behavior but also similar transition times for the two AC input pulse starting points in several simulations.

### 3.4. Refractory Period

**Figure 6:**
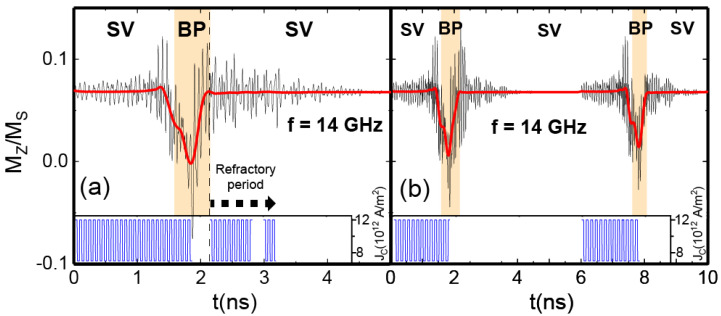
Evolution of the average axial magnetization of the NW when applying oscillatory spin currents of frequency 14 GHz and a magnitude of 5 × $10^{12}$ A/m^2^ upon continuous spin currents of magnitude 7 × $10^{12}$ A/m^2^. The insets represent the input spin-current pulses by means of the applied current. The smoothed red curves are obtained by means of FFT filtering the high-frequency oscillations. Different magnetization states are identified by colors. In (**a**), the input spin current pulses are applied again once the system reaches the SV state. Then it turned off at 2.8 ns and on again at 3 ns (still in the refractory period). (**b**) Displays two consecutive firings of the neuron. The second firing occurs after the refractory period has elapsed and the system has relaxed again into the SV state.

The basic LIF concept can be extended by adding a refractory period in which the neuron is unresponsive to new inputs, mimicking the biological neuron’s brief recovery phase after firing an activation potential. In the case of our NW-based AN, this is accomplished after firing in the BP-DW state and switching again to the SV for 1 ns, as shown in Figure 6a. We also checked that turning off (at 2.8 ns) and on (at 3 ns) the input pulses within the refractory period did not have any response from the system. Once enough time has passed and the system has relaxed correctly, it can be restarted, and the same process may be accomplished again. Figure 6b shows this. We can determine that the refractory period in our NW-based AN is between 3 and 4 ns.

## 4. Device Implementation

We believe that the studied AN holds promise for applications in neuromorphic computing, whether as a standalone neuron system or through the integration of multiple NWs in an array as an ANN. Below, we suggest a possible device implementation of the investigated numerically AN.

**Figure 7:**
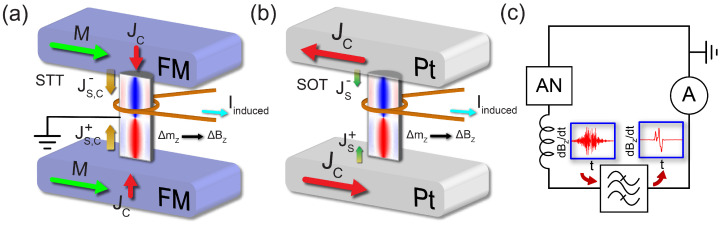
The schematic of the proposed device is designed to implement the described transition and detection mechanisms. (**a**) Electric current is applied to two polarized ferromagnetic electrodes, triggering spin and current injection on the NW via STT and switching from the SV to the BP-DW state. A contact is positioned in the center of the NW to act as a current sink. (**b**) Electric current is applied simultaneously through two Pt electrodes, contacting the opposite ends of the NW in the SV state. This triggers two opposing spin-polarized currents through the NW via SOT, effectively transitioning the NW to the BP-DW state and inducing an electric current to the coil due to the variation of the axial magnetic field. (**c**) Sketch of the detection circuit, incorporating the AN, the coil, a band-pass filter that filters the signal of the induced electric current, and an ammeter to detect it.

To induce the spin current flow, two heavy metal electrodes, such as Pt, can be positioned at the ends of the NW. These electrodes allow to inject opposing spin current flows using two alternative mechanisms: spin transfer torque (STT) with spin polarized electric currents flowing through the NW or spin orbit torque (SOT) provided by interfacial spin-orbit coupling (SOC) with electric current flowing only through Pt electrodes as illustrated in Figure 7a,b, respectively. Using SOT in the device instead of spin transfer torque (STT) could prevent joule heating and Oersted field inside the NW (see Appendix C for an estimation of the maximum working time due to joule heating in the device using STT). Also, more efficiency is expected in SOT-based devices in comparison with devices that perform the spin injection via STT [30]. A coil surrounding the NW will induce a charge current originated from the magnetization changes during the SV-to-BP-DW topological transition and associated fast variations of the magnetic field in the axial direction. We advocate for a detection method focused on capturing the derivative of the axial magnetic field over time due to the small, yet noticeable, magnetization changes between the two stable states. Transition to the BP-DW state will manifest between the two most prominent peaks of the electromagnetically induced current (see Figure 7c). Any oscillations in the electric current could be mitigated through the implementation of a band-pass filter.

Let us briefly discuss the advantages and disadvantages of the proposed spin-based AN in comparison with other recently suggested artificial neurons based on nanomagnets or spintronic devices. First, the most important benefit is that spin-based switching (when using SOT to create spin currents) could potentially have reduced joule heating with respect to the direct resistive-switching-based neuromorphic computing [4]. Secondly, using the excitation of specific spin wave modes to displace the Bloch point (although this perturbation still has to be investigated in more detail) is expected to be more efficient than using a broadband excitation [18]. Finally, it should be easier to tune the dynamic characteristics of the BP in the nanowire-based AN (playing with aspect ratio; see Appendix A) than in a skyrmion-based AN, where dynamics are mainly given by the material parameters [14].

One possible approach for integrating several ANs in the form of arrays is through the utilization of 3D NW networks [12,13,31]. The advantage of employing NW networks fabricated via direct electrodeposition lies in their low cost of fabrication and easy access to the NW ends to deposit the Pt electrodes, which provide the SOT. Additionally, these networks offer the flexibility to tune interwire distances to minimize their dipolar coupling, along with the possibility of using built-in interconnections to facilitate the signal transmission between the ANs.

Potential limitations of the proposed AN include: (i) reduced stability of the BP-DW state owing to thermal fluctuations once the energy of the pulse needed to switch between BP-DW and SV states is reduced; and (ii) the need to amplify the signals transmitted between ANs in the array. Nonetheless, several works have demonstrated that tuning the geometry of the NW surrounding the Bloch points, or DWs, may facilitate the stability of these topologies [18,21,24], which could be a viable way to overcome thermal fluctuations. Additionally, we have found that the input DC current baseline could indeed be reduced with the integration of multiple input signals (see Appendix D), which is useful for efficiency purposes, and to reduce the Oersted field and joule heating in the electrodes of the proposed device.

While manipulating the aspect ratio of the NW allows the tuning of the energy gap between SV and BP-DW states (see Appendix A) and the critical spin current densities employed in our device are close to those used in practical applications [32], SOT experiments usually switch relatively thin ferromagnets (or antiferromagnets [33]), changing their magnetization direction or facilitating the movement of DWs [34,35,36,37]. To our best knowledge, SOT has not been implemented so far to move DWs in longer NWs, as ferromagnets have short spin diffusion lengths [38]. To verify this possibility in short NWs (with the geometry and with spin current density actually explored), we have applied opposite spin currents only within 10 nm thick narrow layers on both ends. While this numerical experiment still has to be optimized in terms of the geometry of the NW used, we clearly observe (see Appendix E) that the BP-DW could be created from the SV state when only interfacial spin currents are used. Before such optimization of SOT-like spin injection is being tuned to decrease the required spin currents, here we propose a similar device in which STT would be involved in the spin injection, with two polarized ferromagnets as electrodes and a current sink in the middle of the NW to prevent the system from failing any conservation laws (see Figure 7a). Here, potential limitations would be the induced joule heating (see Appendix C) and the Oersted field created in the NW due to the injected electric currents. Experimental studies are planned to choose between two suggested options.

## 5. Conclusions and Outlook

In conclusion, we have demonstrated that short ferromagnetic nanowires (with a length of twice the diameter), where the energies of two topologically different magnetic states (Bloch point and single vortex) are close, could be used to create a versatile device for neuromorphic computing. The stable functionality of the suggested artificial neuron has been verified through testing its functionalities, such as integration, leakage, firing, and having a refractory period.

One significant advantage of magnetic nanowires, compared to other proposed magnetic nanostructures for serving as artificial neurons, is the availability of expertise in growing self-organized arrays of cylindrical ferromagnetic nanowires using templates [39,40]. Such AN arrays would also facilitate large areal density, interconnections between ANs, and input (output) communication. These advantages in crafting ANNs using ferromagnetic NWs are in contrast with the difficulty and requirements needed to fabricate arrays of ANs based on magnetic tunnel junctions, memristors, or skyrmions [10] via nanolithography, which could stimulate the development of the ANs proposed here.

## Figures and Tables

**Figure A1:**
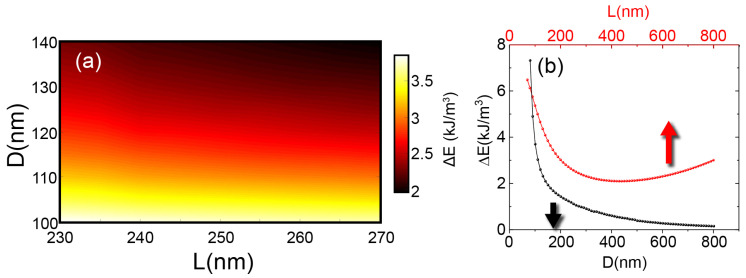
(**a**) Colormap of the energy density gap between the two metastable states for small variations of diameter and length (20 nm more and 20 nm less than the studied NW). (**b**) Energy density difference between the SV and BP-DW states fixing the length at 250 nm and varying the diameter (red curve), and fixing the diameter of the NW at 120 nm and varying the length of the NW (black curve).

**Figure A2:**
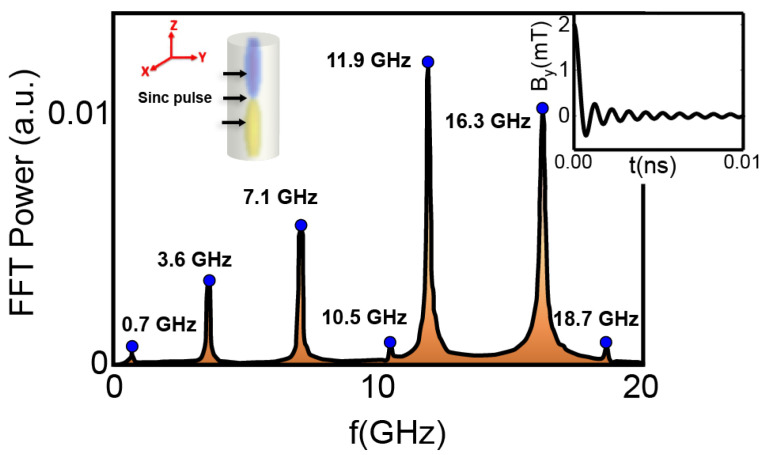
Spin wave modes of the 250 nm length NW investigated in the BP-DW magnetization state. Inset shows the direction of the applied microwave magnetic field sinc pulse to excite the spin eigenmodes of the system. Inset shows the shape of the microwave pulse up to 0.1 ns.

**Figure A3:**
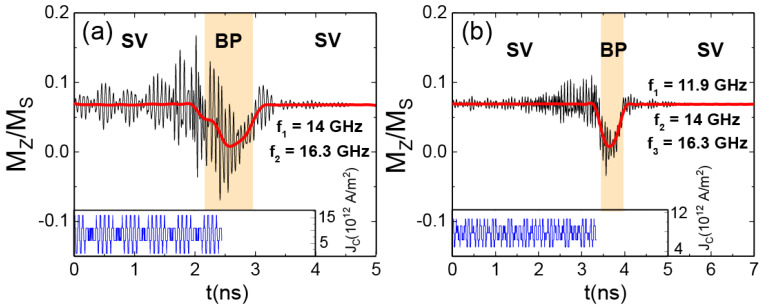
Evolution of the average axial magnetization of the NW when applying the sum of (**a**) two weighed oscillatory spin currents of frequencies 14 and 16.3 GHz and (**b**) three weighed oscillatory spin currents of frequencies 11.9, 14 and 16.3 GHz. The insets represent the input spin-current pulses by means of the applied current. The smoothed red curves are obtained by means of FFT filtering the high frequency oscillations.

**Figure A4:**
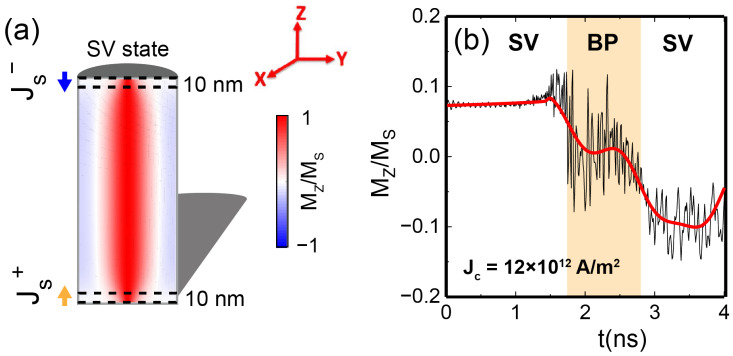
(**a**) Sketch of the initial SV state under the application of opposite SCs in a 10 nm region at the NW ends. (**b**) Evolution of the average axial magnetization of the NW when applying continuous spin currents of magnitude 12 × $10^{12}$ A/m^2^ at the NW ends. The smoothed red curve is obtained by means of FFT filtering the high frequency oscillations. Different magnetization states are identified with colors.

## Data Availability

The data presented in this study are available on request from the corresponding author. The data are not publicly available due to privacy.

## Appendix A. Magnetic State Stabilization with Changes in the Aspect Ratio of the NW

We investigated the energy difference between the two metastable states. This type of analysis is crucial in order to discuss the viability of the state transition.

Figure A1a illustrates the energy density difference map, mapping diameter versus length for the FeCoCu NW under study, with variations of up to 20 nm. Generally, minor diameter changes towards larger diameters tend to stabilize the BP, resulting in smaller energy differences. Also, small length variations towards larger NWs diminish the energy gap. We selected a length of 250 nm for the analysis due to its sufficiently small energy gap and computational efficiency.

Then, we analyzed the energy of the system with significant length and diameter variations, maintaining the diameter at 120 nm and the length at 250 nm respectively (see Figure A1b). For very large diameters the energy gap diminishes to almost zero (up to 800 nm). However, one has to be careful with such small energy gaps due to the possible thermal fluctuations in order to replicate the neuromorphic system experimentally. In the extreme limit of large diameter, the BP-DW will no longer be physically confined and the cost of energy to create it will substantial or even there will eventually be a scenario in which this state is unstable. Below 80 nm diameter, the BP-DW is also not a stable configuration. For substantial lengths, the energy gap reveals a dip for short NWs (200–450 nm), however, this is quickly reestablished and the gap grows for larger lengths. Eventually, multiple BP-DWs emerge of as a more stable configuration compared to a single BP-DW.

## Appendix B. Eigenmodes of the NW in the BP-DW State

The main spin wave modes of the BP-DW state in a 250 nm FeCoCu NW are investigated (see Figure A2). We found the typical low frequency gyrotropic mode (0.7 GHz), and other higher frequency azimuthal modes [18]. The most intense mode corresponds to 11.9 GHz, as expected. This frequency range is used in our input spin current pulses to make the SV to BP-DW switch.

## Appendix C. Estimation of the Maximum Working Time the Case of Implementation in an STT-Based Device

Here, we discuss the possibility of using a STT-based device for the implementation of the spin currents in our AN, as opposed to the SOT-based approach taken in Section 4. This implementation comes with the inconvenience of generating Oersted field and Joule heating inside the NW due to the charge current associated. The maximum working time of the system due to the Joule heating on the device may be calculated in this case.

The heating rate with the corresponding change in temperature over time has the following form assuming a current density J, an uniform conductivity σ and an initial uniform temperature distribution T [41]:

(A1) $$\frac{dT}{dt}=\frac{J^{2}}{C\rho_{m}\sigma }$$

where *C* is the specific heat capacity, σ denotes the electrical conductivity and $\rho_{m}$ represents the mass density.

Since we are using Fe_28_Co_67_Cu_5_ NWs, typical values for this material are used (*C* = 450 J/kgK; σ = 2 × $10^{7}\;\Omega$m^−1^; $\rho_{m}$ = 8.5 × $10^{3}$ kg/m^3^). Thus, for the currents used in our simulations, we obtain: dT/dt = 13 K/ns for the J_*c*_$=10^{12}$ A/m^2^ limit, and dT/dt = 1000 K/ns for the J_*c*_$=10^{13}$ A/m^2^ limit.

As the Curie temperature of the material is approximately $T_{C}=$ 1000 K, we can estimate the maximum working time as $\tau_{max}=T_{C}{\left(dT/dt\right)}^{-1}$. Therefore, for J_*c*_$=10^{12}$ A/m^2^, $\tau_{max}$ = 80 ns and the minimum frequency $f_{min}$= 0.01 GHz. For J_*c*_$=10^{13}$ A/m^2^, $\tau_{max}$ = 1 ns and the minimum frequency $f_{min}$= 1 GHz.

We also notice that if one is able to decrease in a small amount the currents applied, for instance by changing the physical geometry surrounding the BP (see for example, Refs. [21,24]) that would induce extra pinning and modify the energetic difference between the two states, facilitating the transition, the maximum working time would not be a problem in our system for very high currents (J_*c*_$=10^{12}$ A/m^2^) as it operates at around 2 ns and the frequency of pulses corresponds to ∼ 10 GHz.

## Appendix D. Neuron Behavior with an Input as the Sum of Various Signals

To verify the validity of our neuron when two -or more- input signals are used, we conducted additional simulations where the input current pulses were composed by the sum of two (Figure A3a) and three (Figure A3b) pulses, each with different frequencies corresponding to SW modes in the system. These kind of inputs would emulate the rather irregular input pulses that neurons receive. In both cases, the switch between the SV and the BP-DW states in the NW was achieved, and the artificial neuron demonstrated the leaky, integrate and fire functionalities.

This is an important validation to further confirm the biofidelity of our NW-based AN proposal, as neurons can indeed receive inputs from multiple synapses simultaneously, allowing them to integrate information from various sources before generating an output signal. Also, this encourages the integration of our NWs into an array with interconnections between them to develop ANNs in the future.

Additionally, we found that integrating multiple pulses into the signal could indeed reduce the DC current baseline, offering efficiency benefits and mitigating the Oersted field and Joule heating in the electrodes of the proposed device (see Section 4). While a constant baseline of at least 7 × $10^{12}$ A/m^2^ was required for single pulses to achieve the switch, the DC baselines in the case of two pulses (1 × $10^{12}$ A/m^2^) and three pulses (4.9 × $10^{12}$ A/m^2^) was much lower.

## Appendix E. Spin Current Injection in a Thin Region of the NW

One of the main limitations for using SOT to inject SCs in the proposed device (see Figure 7b) is the short spin diffusion lengths of ferromagnets, usually in the range of ∼10 nm [38]. This would indeed be an added difficulty for the implementation of the AN with this particular mechanism, since the center of the NW is at 125 nm (although this can be slightly tuned to obtain a different energy gap between states, as displayed in Appendix A), and interacting with the DW at this scale would be challenging.

In order to verify the applicability of the SOT effect to create the BP-DW here we implement the spin currents only at NW ends within a small (10 nm thick) regions, which are comparable to the spin diffusion length of the system (see Figure A4a). Our numerical experiment demonstrates that, although the magnitude of the spin currents and times of the firing in the AN could still be optimised, the system can indeed switch from SV into the BP-DW state by the influence of the injected spin currents on the much thinner region (Figure A4b) than used in the main manuscript. A continuous spin current with an associated electric current density of J_*c*_ = 10 × 10^12^ A/m^2^ was applied for 4.5 ns in the thin region. The BP-DW state was able to stabilize for more than 1 ns under these conditions, followed again by the switch into the SV state with an opposite magnetization polarity.
